# Utility of instant messaging application, WhatsApp, as a tool to augment post-graduate radiology education

**DOI:** 10.1186/s12909-024-05762-y

**Published:** 2024-07-23

**Authors:** Sohail Ahmed Khan, Shahid Shamim, Waqas Ahmed Farooqui, Rooha Sultan, Munizha Nisar, Noreen Adnan, Ibrahim Khan, Hina Andani

**Affiliations:** 1 Dow Institute of Radiology, Dow University of Health Sciences, University road, near suparco chowk, Karachi, Pakistan; 2 Medical Education and Faculty of Surgery, Aga Khan University, Karachi, Pakistan; 3 School of Public Health, Dow University of Health Sciences, Karachi, Pakistan; 4 Jinnah Medical and Dental College, Karachi, Pakistan; 5 Dow Institute of Health Professionals Education, Dow University of Health Sciences, Karachi, Pakistan; 6 Saint Peters University Hospital, New jersey, USA; 7 Dow Institute of Radiology, Dow University of Health Sciences, Karachi, Pakistan

**Keywords:** WhatsApp, FCPS radiology residents, Active and non-Active contributors, Attitude, Radiology education

## Abstract

**Background:**

Smart phone technology including different instant messaging applications like, WhatsApp, can be used for the development of radiological skills, reporting, and performance. To determine the utility, attitude, and outcome of WhatsApp for augmenting education in FCPS radiology residency program. To assess the opinion of radiology residents regarding WhatsApp as a tool to enhance postgraduate training.

**Methodology:**

A mixed method (qualitative and quantitative) was conducted at Dow Institute of Radiology, Karachi, Pakistan. All FCPS Radiology residents were given a radiological case by principal investigator followed by residents’ response in 24 h. Key findings were shared by the mentor. Before and after the intervention of WhatsApp, all residents were evaluated with written and radiological imaging reporting exam. For quantitative analysis, a closed ended questionnaire was used containing information about total number of messages, images, webpage links shared, level of contribution (active/non-active), and utility (contribution in education related topic only). A feedback form with Likert scale was also got filled by all residents. For qualitative research, semi structured interviews (SSIs) were conducted.

**Results:**

Median number of total images shared were 293 (IQR 1002 images), messages shared 110 (IQR), webpages shared were 54 webpages (61 webpages) and total contents shared by participants was 243 (544 contents). Active contributors showed better performance in utility, competency of contents and attitude towards using social media as a medium for learning. Comparison of written and OSCE results showed better performance after the intervention. Feedback form with Likert scale revealed that students responded positively regarding the shared learning content. Thematic analysis showed 52 codes and 16 themes.

**Conclusion:**

In this research we have observed that WhatsApp is highly efficient and productive academic tool which can amplify postgraduate radiology education. Student’s narrative reflects that residents have found the missing link which can take them to radiological professional excellence through targeted high-profile learning outside lecture hall in time and place convenient motivational environment. Once it will be blended with existing teaching strategy, it can prove to be a game changer.

**Supplementary Information:**

The online version contains supplementary material available at 10.1186/s12909-024-05762-y.

## Background

In the last few years, usage of smart phones has revolutionized many aspects of life establishing the architect of communication and innovation. Smartphones are an essential part of today’s life. It has connected common man and professionals. These days the younger population has been enormously immersed and affected by the social media, so that they can be called ‘digital natives’ [1]. The utilization of smart phones has dramatically increased in general population and exhibit exponential increase in college and university students over the years. Therefore, it is easy to understand that message applications usage is becoming more common than emails in students.

Social media can be simply defined as dynamic and interactive online connection which has become extremely famous in quick time not only for the common man but also for the undergraduate and post graduate medical students including radiology residents and radiologists. Utilisation of credible social media may produce highly efficient and competent radiologist through utilisation of protected theoretical information and images pertaining to radiology [2].

Social media is an important feature of our daily life, its role in the medical education has been exponentially increased. It has many features which has led entry in the corridor of radiology education, however it is underutilised in recent practice. There is certain variability between faculty and trainee utilisation of social media and their imagination [3]. This important point must be considered.

Around 80% of physicians, residents and medical students utilise smart phones and their number is on continuous rise [4]. Current utilization of mobile phone technology of smart phones has provided multidimensional approach for dealing with health care problems. It provides fast and uncomplicated passage to health care problems [5]. Its usage ranges from diagnosing and treating patients up to provision of medical education.

### Types of social media

In recent era conventional means of teaching are being gradually replaced by technology-based learning practices. Twitter and Facebook are doing fabulous job by disseminating highly productive ‘pearls’ of knowledge [6]. Facebook is one of the most initial and effective means of social communication and educational dissemination. It imparts collaborative learning, evaluation, sharing, establishing communication and organisation [7]. Edmodo is another learning application which is protected and cheap in which a teacher has a password which allows a student to join the learning activity outside the classroom [8]. Telegram is an effective way to communicate knowledge through videos audios and notes [9]. Twitter provides small episodes of information which is dependent on liking and disliking of an individual, it can be modified by the users [10]. It’s the duty of the user to verify the shared information from the contributors [11]. Podcast uses chain of digitally downloadable audios which can be used for educational purposes. It can impart knowledge-based content and promote research [12]. It also leads to better memory retention [13]. Blogs are theoretical knowledge in the form of written text on a website. In comparison to scientific journals these are less organised and not strictly structured [14]. Skype can be utilised for medical education through video conferences or tutorials [15]. You tube uses videos and lectures for enhancing medical education, it is dependent on the authentic and credible source for success [16]. Instagram lacks adequate data; it uses educational content including videos which can be seen many times [17]. Reddit is simply a website which is effectively utilised in the preparation of USMLE part 1 [18].

### WhatsApp

WhatsApp is a free of cost application which can be used to share audio and video, files Photos and text messages. It can also be used for effective contact between Pupil and mentors for pure learning purposes. It is one of the most effective social media applications in current practice. Recent research has summed up that mobile phone learning can be a very decisive learning tool in addition to lectures or other classroom teaching strategies [19]. It is easily available and can be used for collaborative learning. Another research concluded that WhatsApp is a very important instrument for dissemination of learning and propagation of curriculum and improvement of education. Some researchers report negative points about WhatsApp, for example it is gender biased and more famous in female students. Few research has also concluded that WhatsApp education leads to superficial learning and distraction [20, 21].

### Significance of the problem

WhatsApp is a commonly used mobile phone application, freely available to all post graduate trainees who are comfortable in using WhatsApp for communicating with their peers and faculty. Learning through such applications can be feasible for busy postgraduate students and their trainers. It can save time and efforts of the teacher and students. In the discipline of Radiology trainees and teachers can use this application to share images and discuss cases with their students, irrespective of time and space. They can also exchange text data, pictures and videos, thereby enhancing the overall depth and breadth of post graduate radiology education.

### Statement of the problem

Radiology education requires multidimentional training of residents. It includes imaging skills, interpretation of images, critical thinking and interprofessional collaboration in discussion. Tools that can provide opportunities to teachers and students for sharing their experience within the limited training time can substantially improve the residency program outcomes. By virtue of its accessibility WhatsApp application has a potential for use in postgraduate training. However, the acceptability and feasibility of using the application is not known in our context. This study will provide basis for possible shift in the teaching learning medium to mobile learning.

In summary, most of these studies revealed contrast between WhatsApp and other conventional learning strategies. The results showed better performance of students in test that were exposed to WhatsApp learning application. Qualitative analysis showed positive attitude, compliance, feasibility, acceptance, and satisfaction of students. Our study is different in this aspect that it has revealed importance of WhatsApp in improving radiology training and imparting comprehensive knowledge.

## Methodology

### Data source

FCPS trainee at Dow Institute of Radiology, Ojha campus of DUHS during June 2022 to August 2023.

### Study participants

All the 16 post graduate trainees enrolled for FCPS programs.

### Study design

This is a quasi-experimental mixed method interventional study in which WhatsApp was used in the educational process of post graduate radiology trainees.

### Study variables

#### Dependent variables

Level of contribution and utility.

#### Independent variables

Age, gender, and year of residency.

### Operational definitions

• CONTRIBUTORS: Any student who did not respond to the electronic post within 12 hours was considered an inactive contributor [22].
• ATTITUDE: A candidate was considered to have a positive attitude if he / she had shown timely response (within 12 hours) and relevant contribution with the intention of serious collaborative learning.

### Data collection method

The study was conducted after getting approval from the institutional review board of Dow University of Health Sciences. All the FCPS postgraduate trainees in the department were given an educational intervention by using WhatsApp application. Quantitative and qualitative data was collected using purposive sampling technique at Dow Institute of Radiology, Dow University of Health sciences, Karachi. All FCPS Radiology residents were included.

### Quantitative data

Single Radiological case including X-rays, ultrasound, CT, or MRI was submitted daily by principal investigator. All residents had 24 h to give their contribution on that case which included giving their pertinent findings, diagnosis, differential diagnosis, and next plan (if any) over that case. Key findings of the case and diagnosis along with the differential diagnosis were shared by the principal investigator. The tutor interacted with every student, pointed out their weaknesses and strengths. Residents were allowed to share their viewpoints supported by national and international research articles and data after 24 h of a post. The same practice was carried out daily for three months. All the data kept stored in the mobile which at the end of three-month time was quantified through a closed ended questionnaire. It contained information regarding the demographic characteristics (age, gender, year of residency), and their interest in the group like total number of messages, images, webpage links shared, level of contribution (active/non-active), competency of content (effective/ineffective), attitude (positive/negative), and utility (contribution in education related topic only or other than education topic or both) will be noted for quantitative analysis.

All residents were evaluated with written test and radiological imaging reporting exam before and after the formation of WhatsApp group to quantify the progress. The standardization was ensured by selecting theory questions and film / spots for film viewing exam / OSCE from past papers and BCQs books for FCPS and FRCR. A feedback form with Likert scale was also got filled by all residents which included questions regarding quality of images provided and benefits of WhatsApp including convenience of time and space and safety of data. Questions were also asked regarding relevance and outcome of this academic exercise in relation to their learning.

Each FCPS trainee data was obtained from the feedback form.

### Qualitative data

Semi structured interviews (SSIs) were conducted with the 16 FCPS students. All students were asked following similar questions and audio recording of all the conversation was done.

i. What is your opinion regarding utilization of instant messaging application, WhatsApp in relevance to your radiological education?
ii. Do you think this mode of education is time consuming in relation to the learning outcome?
iii. Do you recommend inclusion of instant messaging application to the postgraduate radiology curriculum?
iv. Is there any mobile application more useful and affective then WhatsApp in enhancing post graduate Radiology education?
v. Is there any social outcome of utilization of educational WhatsApp group?

The recorded SSIs were then transformed into written transcripts which were read multiple times to figure out the similarities and dissimilarities. Multiple codes were detected from transcripts and from these codes multiple themes were derived.

### Statistical analysis

#### Quantitative

Data from the utilization chart and feedback forms were analysed using descriptive and inferential methods using IBM SPSS Version 27 software (IBM Inc., Armonk, NY, USA). Age in years is given as mean and standard deviation". Frequency and percentages were calculated for gender, and year of residency. Median score of WhatsApp utility (total number of messages, images, and webpage links) compared by level of contribution (active/non-active) using Mann–Whitney test. Written test which consisted of best choice questions (BCQs) along with radiological imaging reporting exam were taken before formation of WhatsApp group followed by another exam with same pattern after three months with completion of research. Comparative analysis of obtained marks (written, OSCE, and total) of residents of different years pre and post intervention was then done using paired sample t test as per data distribution. A p-value of 0.05 or less was taken as significant.

#### Qualitative

The data from audio recordings of semi structured interviews (SSIs) was transformed into written transcripts, handwritten notes were then made from these transcripts and similarities and dissimilarities were thoroughly observed. This is followed by detection of multiple codes from transcripts and from these codes multiple themes were derived. These were organized for analysis. Key themes evolving from the discussions had undergone further explanation.

## Results

### Quantitative results

A total of 16 residents participated in the study of which one was male. The mean age of the participants was 29 years ranges from 26 to 35 years. Most of the students were in their 4th year of residency (*n* = 6, 37.5%), followed by 2nd year (*n* = 5, 31.3%) (Table 1).

**Table 1:** Descriptive statistics of demographic characteristics

Characteristics	*N* = 16 (%)
**Age (years),**	
Mean ± SD	29.3 ± 2.5
Min – Max	26—35
**Gender**	
Male	1 (6.3)
Female	15 (93.8)
**Residency**	
R1	1 (6.3)
R2	5 (31.3)
R3	4 (25.0)
R4	6 (37.5)

*SD* Standard deviation

Active students had a high median score in all utilities compared to the in active students. Active and in active students had a median total content of (548 vs 103), total messages (125 vs 102), total images (488 vs 4), total webpages link (65 vs 12). The results were found significantly different (*p* < 0.05) for the total messages, images, and weblink (Table 2).

**Table 2:** WhatsApp Utility (content, messages, images, & weblink) Scores by Contribution

Utility	In active (*N* = 4)	Active (*N* = 12)	*P*-value^¥^
**Total Content**			
Median (Q1 – Q3)	103 (50—123)	548 (230—915)	0.060
Min—Max	11—129	176 – 2937	
**Total Messages**			
Median (Q1 – Q3)	102 (85 – 108)	125 (104 – 141)	0.004
Min—Max	72—110	100—184	
**Total Images**			
Median (Q1 – Q3)	4 (3—5)	488 (218—1117)	0.004
Min – Max	2—6	11—2632	
**Total Webpages Link**			
Median (Q1 – Q3)	12 (7—15)	65 (48—85)	0.004
Min—Max	5—15	40 – 191	

^¥^Mann–Whitney test

We found the same contributor students results in terms of positive (*n* = 12) and negative attitude (*n* = 4).

Post intervention we found a higher mean score in written and OSCE tests. Pre and post WhatsApp intervention, mean score was found for written test (24.8 vs 36.4), OSCE (34.3 vs 62.6), and total (59.1 vs 99.0). All test scores result was significantly mean different (*p* < 0.001) (Table 3).

**Table 3:** Pre and post WhatsApp intervention mean differences of test scores

Tests (Max)	Pre		Post		*P*-value^€^
	Mean ± SD	Median (IQR)	Mean ± SD	Median (IQR)	
Written (50)	24.8 ± 4.2	25.0 (22.3 – 26.0)	36.4 ± 4.7	38.5 (31.8 – 40.)	< 0.001
OSCE (100)	34.3 ± 9.5	36.8 (23.8 – 39.9)	62.6 ± 12.5	67.3 (52.3 – 70.8)	< 0.001
Total (150)	59.1 ± 11.6	59.8 (49.0 – 68.3)	99.0 ± 16.5	104.3 (86.4 – 109.9)	< 0.001

*OSCE* Objective Structured Clinical Examination (Film viewing and reporting); ^€^Paired sample t-test

**Fig. 1:**
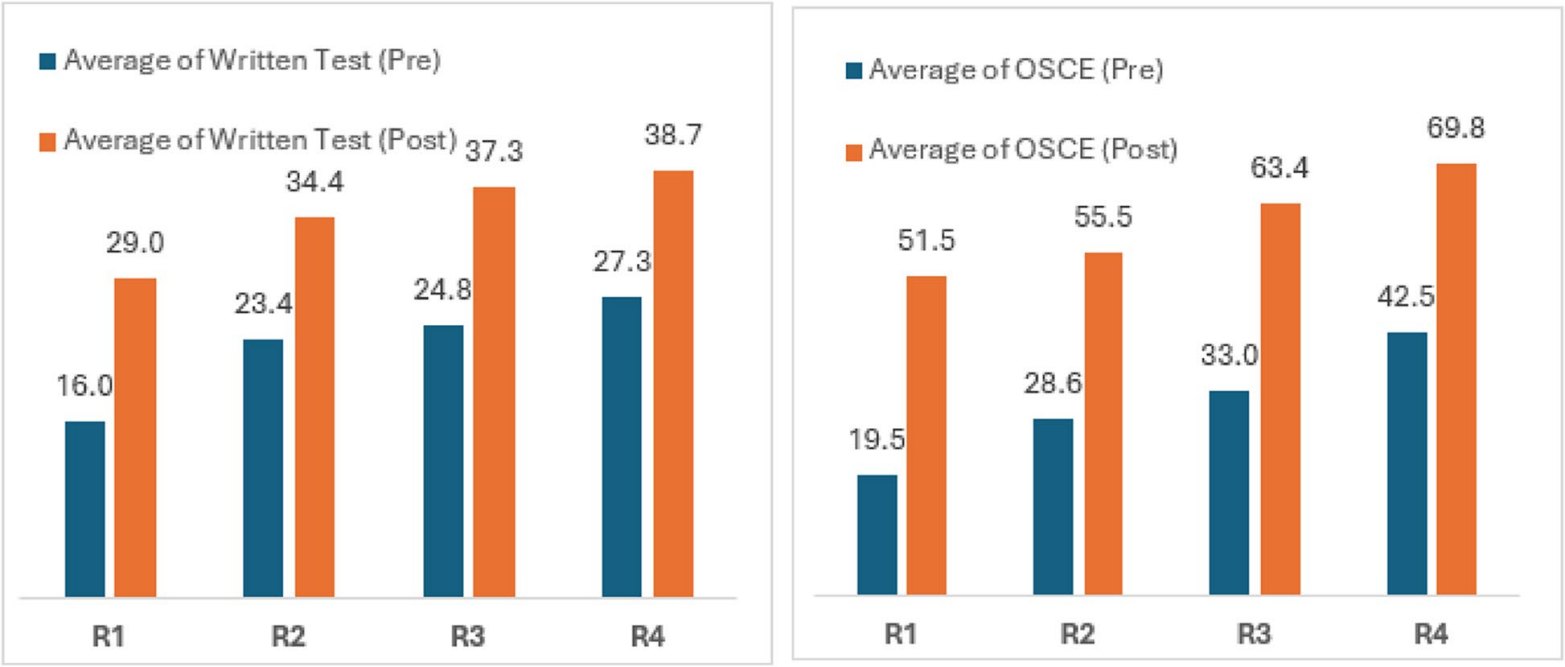
Written & OSCE test pre and post intervention scores among residents

Post intervention we found a higher mean score in written and OSCE tests in all four years of residents (Fig. 1).

All students marked on the feedback for the cases presented. Median scores of the different component of structure and didactics computed and average score found towards complete clarity of objectives. The presented cases were practically relevant to the course and all of them agreed. Participants were also asked on the WhatsApp miscellaneous questions like easy sharing of educational material, time and space compliance, multi-modality, cost benefits, ensure privacy, all of them responded towards strongly agreed (Table 4).

**Table 4:** Cases feedback median scores

Cases Feedback	Median (Q1 – Q3)
**Structure & Didactics**	4.5 (4 – 4.9)
Clarity of Question	5 (4.3—5)
Clarity of objectives	5 (4—5)
Rate the resolution of image	4 (4 – 4.8)
Rate the image quality	4 (4 – 4.8)
**Miscellaneous**	
Easy sharing of educational material	5 (5 – 5)
Time and space compliance	4 (4 – 5)
Multi-modality Learning	5 (5 – 5)
Cost Benefits	5 (4 – 5)
Ensure Privacy	5 (4 – 5)

### Qualitative results

Thematic analysis was conducted, total 52 codes (supplementary file) and 16 themes were extracted from the transcripts which were in turn obtained from interviewing 16 students.

### Themes

#### User-friendly

All participants found it easy to use WhatsApp as narrated by students,

“It’s a user-friendly mode of communication that doesn’t need any subscription or monthly charges, so it’s a free of cost learning tool” (student 1)

“This application is very quick, easily accessible to everyone and can be used comfortably anywhere. We don’t need any system or proper place to use it” (student 2)

#### Sharing of classical exam-oriented cases

Students had the prime opportunity to see classical radiological cases as explained by the student,

“One of the greatest advantages of this platform is that we saw all the scrutinized and selected exam-oriented cases of complexity that has definitely boosted our confidence” (student 3)

“This WhatsApp group has shown lot of cases that I have never seen before in my residency” (student 4)

#### Learning in peaceful environment

Residents are usually stressed out working in the department, however using WhatsApp outside department they are calm.

“We are very stressed sometimes when we are in the department, but in this practice which we are doing on our mobile phone on WhatsApp anywhere, our mind is relaxed, and this is where thinking process starts” (student 5)

#### Increase memorization of cases

Repetition of opinion about abnormality on WhatsApp group has led to increase memory retention, this opinion is backed by student.

“Repeated observation and opinions regarding same topic and its related abnormalities has helped us in effective memorization of that disease” (student 6)

#### Learning art of reporting

Structured radiological report contains pertinent findings, diagnosis, differentials, and management plan. A student told that,

“It adds to our reporting skills, in the end sharing the masterpiece of the final report by our supervisor adds glitter to the gold. It’s a well-organized, authentic piece of work which tells perspective of physician along with management plan” (student 7)

#### The apprenticeship model of teaching really augments learning

One to one interaction between mentor and student is challenging, but it really paid off, one of the candidates said,

“It’s really great when our mentor individually assesses, interact and responds, correcting errors of every resident” (student 8)

#### Going through relevant research articles adds global perspective to learning

It really added golden touch to the discussion, one of the candidates elaborated,

“At the end of the discussion, multiple international articles of different journals are being shared, in this way we can keep in touch with updated and upgraded radiological knowledge" (student 9)

#### Sharpening of radiological eye

Repeated exposure to cases leads to formation of radiological eye, a student explained this,

“This group also helped us in getting our eyes tuned; we are seeing CT, MRI, and X-rays on daily basis. Through this drill I have built an approach, so that I would not miss anything” (student 10)

#### Retrievable data can be beneficial in future

Every case or discussion is saved for future, a student claimed that,

“In future when we will become consultant, this forum would definitely help us in our professional practice, like I can retrieve stored data having similar findings to a particular case” (student 11)

#### Similar on call WhatsApp group should be made

Similar group can be utilized in solving emergency cases, one of the students recommended,

“Through this application we can comfortably contact our seniors. If i am stuck with a case on my emergency call, I can seek his / her guidance. It will be very helpful” (student 12)

#### Junior residents of the group are more beneficiary

One of the senior residents commented,

“This is highly beneficial platform for my juniors who are in year 1 or 2 of training and have less exposure, that they can see exam like cases” (student 13)

#### On comparison with other social media applications, WhatsApp is well supervised and disciplined

One of the senior residents concluded “I have been part of such groups; they are not supervised, and you don’t receive any feedback to improve upon. (Student 14).

#### Time convenient learning strategy

One of the candidates conveyed, “With this application we can study every case in our free time with great ease” (student 15)

#### This should be included in the curriculum twice or thrice a week

One of the residents suggested, “This should be included in the curriculum, but it should be modified, twice or thrice a week would be enough” (Student 16)

#### Improve relationships among residents and with their mentor

One of the students affirmed that, “My interaction with mentor and other students have enhanced due to frequent contact, previously I was under confident, but now I can discuss anything easily” (Student 3)

#### Change in attitude

This academic exercise has brought noticeable change in overall attitude, a resident accepted, “This group has put up pleasant stress which kept us alert and motivated and positively transformed us for multitasking, time management and discipline.” (Student 2).

## Discussion

### Quantitative analysis

In this research we have observed that WhatsApp is highly effective and productive academic tool which can reinforce postgraduate radiology education. Students contributed enthusiastically through answering the questions, images, weblinks and research articles. Student’s narrative reflects contentment regarding this educational tool. Students believed residents had targeted high-profile learning outside classroom in time and place convenient motivational environment.

In our research median number of total messages, images, web pages were higher in comparison to the research conducted by Raiman L et al. in 2017 [23]. This is likely since they included medical students on clinical attachment which may not be that much serious, targeted or lacks motivation. Our study belonged to radiology postgraduates which is all about imaging and students were more serious contributing in a highly motivational environment.

According to our definition of discriminating between active and inactive contributors, four of the students were found to be inactive and the rest were all active.

In our research we found that the contribution of the students showed a significant increase from the first month to the second month and it reached its maximum in the third month. This incremental pattern refers to the increased interest of most of the students in the group. However, the inactive contributors showed some decline in the second month and again show a slight increase in the third month that also showed the pattern of increase contribution, however, in the end it was not statistically significant.

We observed that there is increased effective utilization of WhatsApp academic group by the active contributors they also showed high competency and highly positive attitude which was shown by their timely relevant contribution for the collaborative learning.

This research also contained an assessment part which is further comprised of radiological imaging reporting exam and theory exam which is all composed of BCQs. These exams were taken before and after the intervention of WhatsApp group which was formed for three months.

Our study has shown that greater marks were obtained by the residents after this exercise both in the film viewing session and theory examination. In the research conducted by Santos GN et al. which included undergraduate dental students, also used MCQs and radiological imaging for oral radiology course. Their study showed an increase in the number of correct answers, but they did not have pre and post intervention comparative analysis [24].

Gon S et al. compared WhatsApp with lectures. They also gave questionnaire pre and post intervention which also revealed increased marks obtained by medical students after WhatsApp intervention, but they have also compared it with marks obtained in lectures [25]. Clavier T et al. did research which included anaesthesia students divided into WhatsApp and control group, they also did assessment with MCQs which showed no change in two groups [26].

In our study there was no considerable difference in exam performance observed of active and non-active participants. The non active participants were the senior most residents. They participated in the group activities, but it was delayed with a lesser number of contributions to the group because few of them had final exams closely aligned and others had a newborn baby to take care of. But even then, they managed to obtain good post intervention marks because they had already spent more time in the department, and they had more radiological exposure.

Our research had shown that highest post intervention means scores were obtained by fourth year residents, followed by third, second- and first-year students. This order is explained by the experience, exposure and time spent in the residency program.

A feedback form with Likert scale revealed that students responded positively with WhatsApp, this is well aligned with the research done by Primmer et al. who did multinational research regarding instant messaging application, they used Likert scale feedback which showed that it is user friendly, and participants were satisfied with it [27]. Vilgrain v. in 2017 disclosed that women's future is highly optimistic in Radiology in France. There is no gender discrimination in Radiology residents. Women opt for Radiology residency as it is an interesting field, consequently academic designations for Radiology are also on rise. In our research there is a heavy predominance of female residents. Lack of males in the medical field and Radiology is due to delayed settlement and earnings, long study years and multiple exams as compared to other fields [28].

### Narrative of qualitative analysis

Sixteen themes were ultimately derived after going through semi structured interviews of 16 residents.

1. User-friendly:

WhatsApp is free of cost, very easy to operate social media application. It is already built in every mobile phone. You only need to have good internet connection.

2. Sharing of classical exam cases:

One of the reasons of success of this group is that students were being exposed to important selected exam like cases. Students saw around 90 exam cases, roughly with one differential, they had covered approximately 180 exam like cases. Yaqoob et al. in 2020 did research on WhatsApp that also showed increase in learning curve of orthopaedics graduates [29].

3. Comfort zone radiology practice:

Students can use WhatsApp at home lying in casual clothes in easy postures, which enhances their focus on posted cases and ultimately their academic output. Choudhry RI et al. did research in 2021 which also concluded that WhatsApp provides time and place convenient communication [30].

4. Increase Memorization of cases:

Once a case is displayed on WhatsApp group, all residents share their findings and articles which really foster memorization of that abnormality.

5. Learning art of reporting:

Students used to comment on a particular case followed by sharing of final comprehensive report by the mentor which contained pertinent findings, diagnosis, differential (if needed) and management plan. Students learned fine lines of art of reporting and variability among reporting of different diseases.

6. Apprenticeship model of teaching really augment learning:

One to one interaction of mentor with the students, pointing out their mistakes with rectification plan really leads to exponential long lasting learning. Nicholsan S et al. did research on instant messaging application that showed appreciation of immediate feedback and response by mentor [31].

7. Going through relevant research articles adds global perspective to teaching.

Every case discussion includes sharing of different international articles about the topic that led to global orientation, lateral thinking and widening of vision about the subject.

8. Sharpening of radiological eye.

It’s simply the early accurate pick of abnormality. Candidates saw cases on WhatsApp group multiple times with multi-modality exposure. Finally, the mentor pointed out their mistakes, this drill led to sharpening of radiological eye.

9. Retrievable data can be beneficial in future.

Ninety cases with their history and structured reporting are the data stored with all residents. In future if the students would see same or similar cases, they can always refer to already discussed stored data. Raiman et al. in 2017 did research on WhatsApp that also revealed saved learning content.

10. On comparison with other applications, WhatsApp is well supervised and disciplined.

Facebook and Telegram are also competitive for academic discussions containing large number of random contributors from many disciplines. This ultimately brought disconnect among participants and many times outcome is not shared. But our WhatsApp group included only departmental radiology postgraduates, all participants have daily interaction including mentor which is impossible in other applications.

11. Similar WhatsApp group should be made for emergency on call radiology residents.

During this research we have found that students wanted to form similar separate group which can ensure supervised team based radiological practice which can aid critical decision making that will ultimately benefit emergency patients.

Jhonston MJ et al. in 2015 did research that revealed WhatsApp to be better effective tool aiding in provision of better monitoring of emergency patients [32].

12. Junior residents are more beneficiary of this group.

By the virtue of this group junior residents were exposed to exam cases at an early phase of their training reducing fear factor in future. Senior residents reported that they did not have similar exposure when they were juniors.

13. Time convenient learning strategy.

All residents agreed that the outcome of this learning strategy is multi fold in comparison to consumed time. Students can even contribute once they are off from their duty timings.

14. This should be included in curriculum, twice or thrice a week.

Almost all students agreed that, since this academic group is highly beneficial therefore it must be included in the curriculum.

15. Improve relationship among residents and with their mentor.

By the virtue of this group all participants have frequent productive daily interaction which otherwise would not be possible due to postings at different stations. This has really reduced hesitation, boosted confidence and formed strong ties among participants.

16. Sharing of motivational quotes transformed positive change in the attitude of students.

Motivational conversation during feel of low emotional and moral status led to enhancement of moral and confidence of students. All this exercise has led to increased contentment among radiology residents.

### Limitation

Since our research was a single centre study, therefore it does not reflect the opinion of radiology department of whole city or country. Therefore, to make practical decisions like whether to make utilization of WhatsApp as part of curriculum or not must need large scale multi centre studies.

## Conclusion

In this research study we have observed that WhatsApp is a highly capable, efficient, and productive academic tool which can amplify postgraduate radiology education. Students’ narrative reflects that residents have found the missing link which can take them to radiological professional excellence through targeted high-profile learning outside lecture hall in time, place and posture convenient motivational environment. Students showed enthusiasm and contentment for this mode of education which has provided supervised education, feedback, improved radiological eye, communication, and reporting skills. Once it is blended with the existing teaching strategy, it can prove to be a game changer.

### Supplementary Information

Supplementary Material 1.

## Data Availability

The datasets used and analyzed during the current study are available from principal investigator (corresponding author) on reasonable request.
