# Supplementary material for: Utility of instant messaging application, WhatsApp, as a tool to augment post-graduate radiology education
Source: BMC Med Educ. 2024 Jul 23;24:789. doi: 10.1186/s12909-024-05762-y (PMC11267667; doi:10.1186/s12909-024-05762-y)
Supplement: Supplementary file 1 — Supplementary Material 1. [file 12909_2024_5762_MOESM1_ESM.docx]

## CODES

1. Free of cost.
2. Easily installed on phones.
3. Quick.
4. No technical hitch.
5. Convenient reutilization.
6. Unique rare diverse case.
7. Biopsy reports are also shared that also consolidate learning.
8. Minimizes fear of difficult cases.
9. Residents see cases daily in very busy and hectic environments while multitasking. However, through WhatsApp they have enough time of 24 hours so that they can comment in a stress-free environment.
10. This learning is convenient in time place and even posture, you can comment even in lying down position at home**.**
11. Repeated hammering on single case through reporting by all resident's help increase in long-lasting memorization.
12. Documenting findings over a case by all residents is followed by discussion over it and on its differential diagnosis. This all has led to increased retention and comprehension of the topic/case/subject.
13. Learning of art of reporting is the key feature of this mode of learning.
14. Initially all residents comment on a single case according to their understanding and reporting styles so students are privileged to see a case through multiple lenses.
15. At the end final craft of report writing had been shown by our mentor, it will give final finished information, which is well structured, organized, concise and contained management plans.
16. Reporting accuracy has been greatly increased due to increase in our radiological vocabulary and word bank.
17. One to one interaction of supervisor with the students on reporting really gave exponential increase to long lasting learning and understanding of subject.
18. The mentor pointed out mistakes directly to students and gave rectification plan for it.
19. Going through research articles in relevance to the discussed abnormality really added an updated global radiological version of that disease and potentiates lateral thinking.
20. Sharing of articles and exposure to national and international literature had really widened the radiological acumen, visualization and understanding in global perspective.
21. There is marked sharpening of the radiological eye due to multimodality exposure each day.
22. Daily placement of cases and presenting same abnormality on different radiological modalities like x-ray, ultrasound, CT scan and MRI. The supervision of mentor by pointing out the missing findings really sharpened the radiological eye which means picking up abnormality and its finding in least quick time.
23. The data is retrievable and can be seen any time if a similar case is seen in our daily practice afterwards.
24. So, this one-time stored data that was kept for this group can be a reference and focal point for future similar cases. One can easily go back to search the data pattern and follow the way of reporting.
25. So that the data shared in this research would be easily beneficial in times to come.
26. There is a suggestion that similar academic WhatsApp groups should be made for on call duty doctors, so that they can put up any difficult emergency case and know the desired information.
27. This will strengthen the practice of radiology among residents, they would be able to see difficult emergency cases which will be resolved by consensus. When similar cases are encountered in future they can attend confidently, and the ultimate beneficiary will be the patient.
28. Junior residents are specifically big beneficiaries of this group due to early exposure to exam cases / difficult cases.
29. Early exposure will make them more expert in future.
30. Their difficulty level will also be reduced in future and their confidence level will increase.
31. WhatsApp is different from other online applications in that it is well supervised.
32. Our academic group contained a smaller number of students, Facebook and telegram contained many students; therefore, no effective individual interaction was possible.
33. Since large number of students are not known, this reduces interaction with other fellow students.
34. Our group contained selective postgraduate radiology students and other applications contained random people from different modalities and walks of life that led to difficult interaction.
35. WhatsApp is more user friendly; it is more productive since it is well supervised containing limited number of known colleagues with direct mentor and student interaction.
36. Other messaging applications like Telegram and Facebook contain large groups in which people don't know each other and there is no direct supervision, and these are not as disciplined as this group was.
37. Time consumed during this educational exercise is far less in relation to multifold learning outcome.
38. Even married women can take out time of their busy schedule at home.
39. In other groups anyone can comment anytime. In our group the order of radiological contribution starts with 1^st^ year, followed by 2^nd^, 3^rd^, and 4^th^ year. Our students wrote their findings in this order so that juniors who have less knowledge should not copy paste senior explanation.
40. WhatsApp group was also time bound in outcome disclosure, while in other applications final diagnosis is not necessarily disclosed.
41. Our group provided outcome-based learning.
42. Other groups like Facebook contain distractors that they do not follow the discipline and at times it contains an irrelevant objectionable content and advertisement.
43. There was consensus among participants that in comparison to the outcome consumed time did not bother them.
44. WhatsApp based teaching learning strategy should be included in curriculum but to do it effectively and consistently it is better to do it twice or thrice a week.
45. This group had really improved relationships between senior and junior colleagues and mentor.
46. Sharing cases and academic content in the group had really boosted the morale of the students and increased their efficiency.
47. Minimizing fear of difficult cases in future.
48. Improved communication skills.
49. Understanding of team based collaborative learning.
50. Increase confidence.
51. Sharing motivational quotes really increased the urge of learning even when the students are tired.
52. All this exercise has led to an increase in happiness and satisfaction among residents and reduced their anxiety regarding studies.
